# Extracts and Gleanings

**Published:** 1876-11

**Authors:** 


### Extracts and Gleanings.

*Local use of Bromide of Potassium. Coomes.* (*Cincinnati Medical News*, February, 1876.)—The bromide of potassium applied to mucous surfaces, is a local anæsthetic, although this effect is secondary unless used in weak solution, say ten to fifteen grains to the ounce of water. The action of the bromide when applied to mucous surfaces in substances, or saturated solution, resembles that of caustic. Its effects upon mucous surfaces are not visible, like those of an ordinary caustic. It does not whiten tissues, nor is its application painless, as is the case with many caustics. When applied to the schneiderian membrane of palpebral conjunctiva the pain is severe and of a hot burning character. The larynx and fauces are more tolerant of its action than the eye or nose, but the pain is similar in being associated with heat. The duration of pain is never more than a few seconds. Applied to congested mucous surfaces, it disgorges the distended vessels and increases the secretive action of the mucous follicles.

In papillary ophthalmia, commonly called "granular lids," the results of its action are similar to those obtained from the use of muriate of ammonia. It reduces the hyperthrophy, increases the amount of secretion, and allays pain. Its anæsthetic properties alone give it an advantage over the ammonia.

In the treatment of nasal catarrh, where there is a dry condition of the membrane, the bromide in powder or saturated solution is an agent of great value. Where there is hypertrophy of the membrane lining the nasal cavities, with an insufficient amount of the normal secretions, a condition met with in proliferous inflammation of the membrane, insufflation of the powdered bromide, or injections of the saturated solution, produce excellent results. By its use the secretions of the membrane are increased, congestion lessened, and a marked reduction made in the hypertrophied tissues. Its immediate effects in these cases of proliferous inflammation of the nasal cavities is to relieve the patient of that sense of "stuffiness," which is most always complained of.

For the last year and a half I have relied almost entirely upon the bromide of potassium as a local agent in the treatment of throat affections. It has but rarely disappointed me. The results which I have obtained from its use in this class of diseases, have been most gratifying. In cases of acute tonsillitis and pharyngitis, it matters not whether in their incipiency or in the advanced stages, a solution of bromide of potassium, sixty grains to the ounce of water, applied with a mop, or with an atomizer, every hour or two, will be found to produce well nigh complete relief. In cases of ulceration, the open sore should be touched with carbolic acid or nitrate of silver. In but few cases will it be necessary to apply the escharotic a second time. Under this plan of treatment, all the painful and distressing symptoms that attend such cases, speedily disappear. In every instance the patients treated with the bromide, expressed themselves as feeling a great relief immediately after the application of the drug. These statements have been verified by the rapid reduction of temperature in the affected part, the restoration of the functions in the mucous follicles in the vicinity, the disgorgement of the distended blood vessels, and almost an entire absence of pain during the whole course of the disease.—*Half-Yearly Compedium*, July, 1876.

*Hydrate of Chlorol to Prevent a Recurrence of Chill and Fever.*—Dr. Lee (*Medical and Surgical Reporter*) having a case of intermittent fever, and wishing to arrest the paroxysm at once, gives the following history:

"In conversation with a highly intelligent clergyman, who had spent many years in Texas, he alluded to the plan adopted by physicians there, in such cases, of giving a full dose of opium in anticipation of the chill, and causing the patient to sleep through the time in which it should occur. I at once determined to give the method a trial, substituting chloral for opium, as being more essentially antispasmodic in its action. Giving quinine as before, about fifteen minutes before the time at which algid symptoms had begun to declare the viously, I caused her room to be darkened, and all unnecessary noises about the house to be stopped; administered twenty grains of chloral hydrate, dissolved in camphor water, and left her. In ten minutes I returned to her room, with the intention of repeating the dose if there were any sign of excitement about her. She was already, however, sleeping quietly, with pulse and skin natural. I visited her at intervals of about an hour, always to find her in the same placid sleep, from which she awoke much refreshed, about 4 P.M.; the chloral having been administered soon after 10 A.M. There was no return of the chills for that season. I, of course, took the precaution to continue the antispasmodic and depurative treatment for a considerable period.

"Seven months later, on the 10th day of May of the present year, she was again seized with a severe chill. Profiting by my experience on the former occasion, I now directed that she should be closely watched on the day of the recurrence, the quinine administered as before, and at the first indication of the invasion (which previously had not been awaited) the usual dose of chloral administered. The person to whom I entrusted the case had been with the patient in all the previous attacks, and was an intelligent and trustworthy observer. At 11 o'clock she noticed a slight blueness of the nails and coolness of the finger tips, and accordingly at once gave the dose. The result was as satisfactory as before. There has, as yet, been no return of the intermittent, although the subsequent treatment was continued but a short time."

*The Management of Diphtheritic Paralysis.*—The eminent Sir John Rose Cormack says on this subject, in the Edinburgh *Medical Journal:*

Iron is particularly indicated in diphtheritic paralysis, as the patients are always anæmic. There are few cases in which its administration does not prove itself in an obvious manner to be useful in a high degree. Sometimes it is only borne in very small doses.

Nux vomica, either in the form of extract or the liquor strychniæ of the British Pharmacopœia, taken daily, with some ordinary combination of laxatives, such as the compound rhubarb pill of the British Pharmacopœia, ought to constitute a part of the treatment in nearly every case. It increases the peristaltic action of the intestine, imparts expulsive and retentive power of the bladder, and likewise has a general influence in improving innervation. The dose ought to be moderate, for large doses prove too exciting to the nervous system, and so tend to exhaust rather than invigorate its flagging powers. From half a grain to two grains of the extract, once a day, with or without the occasional or constant addition of from five to ten drops of the liquor strychniæ two or three times a day, are suitable doses.

Local treatment is of the most importance with a view to direct toward the wasted and wasting muscles a greater supply of blood, and thereby improve their nutrition. Occasional blisters act very beneficially in this way; but they must not be relied on to the exclusion of the constant use of stimulating pastes or liniments. I do not know of any local stimulant more efficacious, or better adapted for continuous use, than a ginger and mustard paste. The object of using the paste is to maintain a warm glow in the skin without vesicating it. The potency of the paste must therefore be proportioned to the susceptibility of the skin. By applying two powerful a stimulant to an extensive cutaneous surface, we may be obliged to suspend the local treatment, and so impede the progress of the cure. In some excitable patients who cannot bear long continued counter-irritation of the skin, a gentle kneading of the paralyzed muscles three or four times in the twenty-four hours will be found useful as a means of directing a supply of bl od to them. In such cases, after each kneading, a moderately stimulating liniment containing a small quantity of laudanum may be applied with great benefit. The laudanum prevents an uneasy bruised feeling, which is often complained of after the kneading, and in irritable subjects is apt to induce restlessness and insomnia.

Galvanic excitement of contraction in the paralyzed muscles is often decidedly useful; but it is a measure which requires to be employed with moderation, and at intervals of about twentyfour hours. If resorted to too early, or too freely, it exhausts the nervous power of the afflicted muscles.

*Liquor Potassæ in Chlorosis.*—In the *Canada Medical Record*, Professor A. P. Reid gives this case:

A. B., aged twenty-four, came under charge at the Provincial and City Hospital, with the following history: Had been admitted about two months previously, under one of my colleagues, complaining of nervous debility; there was amenorrhœa, and the ordinary symptoms of chlorosis. The recognized means of relieve had been judiciously used, without any benefit; in fact, the house-surgeon said she was worse than on entering.

Examination showed that there was no recognizable disease of the heart, lungs, kidneys, stomach, or liver, but amenorrhœa strongly pronounced, anæmia and impoverished blood, venous hum and anæmic cardiac murmur, and general anasarca, which simulated the last stage of Bright's disease, with inability to even to sit up in bed.

I concluded that the best tonic or alterative would be liquor potassæ, as it excels all other diuretics in the amount of solids carried away by the kidneys. Its use was contra-indicated from its known effect of producing debility and watery state of the blood when long continued, and as well of impairing digestion. Evidently, however, the patient would not hold out unless releived speedily, and the liquor potassæ was given a trial—ten minim doses in mucilage three times a day.

In the course of two days the very swollen condition of the legs was a little ameliorated (no bandaging being used), and the appetite was, if anything, better.

This improvement continued, and in the course of two weeks she was able to set up, the anasarca having quite disappeared. The cardiac murmur was lessened, and the pasty color of the skin was a little relieved.

In three weeks' time the liquor potassæ was discontinued; she had become very well, and was able to leave the hospital in five weeks quite restored, milk and nourishing diet, with liquor potassæ, being the only means used.

Since then I have frequently resorted to this drug in anæmic amenorrhœa, and, with a few exceptions, with very great satisfaction, and in no case have I seen it productive of injury.

In considering the course of chlorosis, we first have retention of the masses of an excrementitious blood, which debilitates, if it does not poison, the assimilating properties of tissues; and if this be the fact, an agent which would stimulate and assist excretion should be the most efficient medicine. Such we have in the liquor potassæ, and to this I attribute its curative power. The blood poisoning being removed, and the assimilative powers rapidly recuperate, without the neccessity for special tonics.

During my last three months' duty at the hospital, every case (six in number) of uncomplicated chlorosis was placed on liquor potassæ, and all got well rapidly, without other medication, unless a laxative when necessary.

*Treatment of Sprain.*—The surgeon, Mr. John Gorham, writes to the *Lancet:*

Mr. Aston Key used to say in his lectures: "When called to a sprain you may apply heat or cold, according to the feelings of your patient; but, on the whole, I recommend hot applications." For thirty years it has been my practice to follow my leader, and to use heat in every case. There can be little doubt that the rest enjoined and instinctively employed by the patient, whether enjoined or not, has much to do with the result in either case, and it is equally true, according to my experience, that by far the best adjuvant to rest is heat and moisture properly applied. I use the "properly" advisedly, because for a long period the topical treatment was neither hot nor cold, but a compound of the two—blowing hot and cold, as it is said, and this from sheer carelessness. A flannel steeped in hot water was used by the advocates of the hot system; lint saturated in cold water by those who adherred to the cold. By midnight, in either case, the application had become lukewarm, and the case might be said to have been conducted through its stages by rest *plus* moisture, regardless of temperature. That the employment of rest was the almost sole and efficient agent in the cure, may be inferred from the labors of Mr. Hilton, who, by his writings, has emphasized this element, rest, as one of the most powerful in the agenda of surgery. But to return. Heat and moisture, if applied in a slovenly way, will mar the whole. For a sprained ankle, take a piece of lint of such size that when folded thrice it shall be four inches wide and twenty inches long—sufficiently wide and long, in other words, to completely envelop the joint; let this be soaked in boiling water, squeezed out gently, and applied to the limb. Next, take a piece of thin gutta-percha shaving or oiled silk, two inches wider than the folded lint, on which it is laid, with a margin an inch wide above and below, which lies in contact with the skin and prevents evaporation. Lastly, over the whole apply a bandage, and tie the limb on a pillow with two pieces of tape.

*Treatment of Remittent Fever.*—Dr. Records, (*St. Louis Med. & Surg. Journal,*) in giving his plans of treating remittent fever, referring to one case, remarks:

11 A. M.—Temperature 105½°, *weighed* out 12 grs. cinchonidia sulph., and gave it at once. (Child five years old.)

12 M.—Temperature 105½°.

1 P. M.—104½°.

2 P. M.—103½°.

4 P. M.—102½°.

5 P. M.—102°.

Here it ceased to descend, for six hours remaining the same, then it began to rise and another dose, same as before, was given, followed in three hours by a complete remission, or rather convalescence, as she had no more fever.

Now from the time she took the first dose, no other medicine was given until convalescence was established.

The next day she got one grain of the medicine every four hours as a tonic, with milk and whisky punch. She also got three very small doses of calomel, ipecac and chalk mixture, three hours apart, followed by magnesia sulph. sufficient to move her bowels.

My plan is to wait until the fever reaches the maximum, then give cinchonidia or quinia, and give more of the febrifuge mixtures usually prescribed, and no calomel unless there is a very dry tongue heavily furred, and then in minute doses, followed by magnesia sulph. or ol. ricini.

With this treatment a large number of doses are kept out of the patient's stomach, which is already irritable enough.

I know that physicians can hardly give up the idea of the small doses of quinine oft repeated, yet whenever one sees the superiority of giving large doses, when indicated by the thermometer once, he will never resort to the plan of waiting for a remission and give small doses for its antiperiodic effect instead of its antipyretic.

*A Useful Mode of Administering Chloroform.*—In the *Medical Times*, a correspondent who writes from London recommends the following simple form of anæsthetic apparatus:

A pleasant, safe and economical method of administering it will be found in placing a handkerchief in an ordinary tumbler, and then dropping some chloroform upon the handkerchief, the tumbler after this being held to the nose. The advantages of such a plan are these: The tumbler cannot be so applied as to entirely exclude an admixture of air along each side of the nose. Then but a small quantity of chloroform is used, for as soon as each pain is over, the tumbler can be placed bottom upwards on a plate, and the loss by evaporation reduced to a minimum. So slowly does the evaporation take place, that the medical attendant can add the chloroform required at intervals himself, and so be sure of the quantity. Further, in emergencies, the patient may administer the chloroform herself, the glass falling from the hand as soon as unconsciousness creeps over her. In this respect this plan is much superior to the old one of giving the anæsthetic on the handkerchief alone, which often kept its place after the hand had lost all command over it. As a means of administering chloroform in ordinary cases, the practitioner will find this plan to possess great advantages, and to secure the patient very safely against danger, as well as to be most convenient and economical. Where it is necessary to intrust the administration to a nurse while the medical attendant is engaged otherwise, by this means all risk is avoided, and occasional supervision is all that is necessary.

[We have habitually adopted the above plan of administering chloroform to parturient women, for the last twenty years, and strongly endorse all that has been said in its favor.—Ed. P. & S.]

*Cholera Infantum.*—Dr. McNider (*Medical and Surgical Reporter*) writes:

My treatment of the affection is simple, and has been much more satisfactory than that laid down by the books. If I see the little patient in the begining of his trouble—and this is very important—and if there is no great urgency, I give one-fourth to one-half grain of calomel, with one-half to one grain of rhubarb, every hour. The calomel with the object of inducing, and until presence of bile is seen in the evacuations. The powdered rhubarb for its secondary astringent effects. If there is much fever, I reduce the temperature and thirst by cold sponging, taking care not to expose too much of the body at one time, and not too cool off too suddenly. Bits of ice or cold water are given *ad libitum.* Cloths frequently wet in cold water are applied to the head. For the almost inevitable nausea and vomiting, I use mustard rubbed up with lard or oil, instead of vinegar or water, applied over the stomach. In this way the mustard is less irritating, and equally as effectual. If this does not control the vomiting, spice poultices wet in brandy are next used. Subnitrate of bismuth internally. I never use the fly-blister upon an infant.

Some practitioners prefer murcury and chalk instead of calomel; but as chalk in all its preparations has disappointed me so often, I never use it, except the pulv. cretæ comp. cum. opii, and this is preferred as a mild anodyne, and for the stomachic and astringent effect of the tormentil in it.

Quinine is my main reliance and this is my plan of giving it. Learning when there is an apyrexia, I give, during this intermission or remission, from a half to one grain of quinine, according to age every hour, *until its effect is seen*, and then continue it once in two hours, until the expected exacerbations, when it is discontinued, to be resumed again at the next apyrexia, and so on. Quinine is doing its duty when it tranqualizes the nervous system, relaxes the skin, and clears off the tongue. Used in this way it is wonderful how much it modifies the next paroxysm. When there are no cerebral symptoms to contra-indicate it, I combine with the quinine from a quarter to half a grain of Dover's powder.

The physician can only feel hopeful when he has succeeded in re-establishing the normal excretions of the liver and kidneys. The persistent use of calomel and quinine will do this. Astringents are called for and demanded. I have used all that are recommended, and have been as often disappointed. Catechu, kino, rhatany, etc., may do very well for diarrhœa, but in cholera infantum, when the inflamation has advanced, say to entero-coleitis, they are ineffectual. In the oxide of zinc I think we have one of the happiest remedies. I have used it so often and with so much satisfaction that I can confidently recommend it.

Not only do we have a weak condition of the nervous system at large in this affection, but particularly so of the nerve centres that control the whole elementary canal; again, from the impressionable state of the nervous system of infants, there is, as we know, a great predisposition to convulsions, and other spasmodic affections, to which cholera infantum is not a little correlated; again, the bloody stools show us that there is a hyperæmia of the excreting surfaces of the canal. These facts demand a remedy possessing not only astringent but anti-spasmodic virtues, and the oxide of zinc is just the article that fulfills the indications. I gave this in two or three-grain doses, every three or four hours, according to symptoms, either alone or with pulv. ipecac. or Dover's powder. If gastritis complicate the affection, I use the subnitrate of bismuth. For the tormina and tenesmus, I use injections of terpid water, and the usual starch and laudanum enemata. Sulphate of zinc (half a grain to one ounce) has acted happily for me, and I have seen no bad effects from it.

For the acidity of the stomach, instead of lime water, I add a few grains of soda bicarb. to the milk or barley water.

Strict attention should be paid to the diet. A child with this affection needs to be well sustained and nourished. Brandy is indispensable.

*The Menses in Phthisical Patients. Ladmiral.* (*Lyon Medic.*, No. 22,)—Menstruation and evolution are two distinct and yet closely allied functions; hense the possibility (and it is not unfrequently noted) of conception by a woman whose menstrual flow has disappeared under the influence of a general disease.

Ordinarily the suppression of the menstrual flow, in a woman who coughs and becomes emaciated, is a symptom which points to the rapid evolution of pulmonary tubercolosis. In a large number of patients the catamenial function is suspeded at the same time that the disorder of the lungs is advancing; there are rarer cases where the amenorrhœa precedes the habitual manifestations of the diathesis. It is this fact which leads some to look to the uterus for a lesion which does not exist. Hence, always when there is menstrual suppression without appreciable cause, and when pragnancy cannot be considered possible, physicians should carefully examine the chest, and give a reserved diagnosis. The menstrual flow, however, has no influence upon the march of pulmonary phthisis; but its reappearance always coincides with a period of arrest or cure of the disease (temporary or permanent), and is a proof of the fair condition of the constitution.

Dr. Molliere, in reviewing Ladmiral's treatise, concludes that the author has not been sufficiently explicit on this point, viz: whether the existence of pregnancy should be admitted or denied when these variations in the menstrual function occur. It is often true that women who are profoundly affected are also apt to conceive; and, *per contra,* certain general symptoms may co-exist with amenorrhœa, and lead to a suspicion of the early stage of a pregnancy which does not exist.

*The Abortive Treatment of Whooping Cough by Sulphate of Quinine. R. K. Hinton, M.D.* (*Med. and Surg. Reporter*, August 26, 1876.)—Dr. H. reports ten cases in which this treatment was successful. The ages of some of the patients are omitted in the report, but we gather that the range of their ages was at least from eighteen months to twenty-four years. The doses given were one to five grs. every three to six hours, according to age and severity. In all the cases the pertussis was aborted, the period required varying from a few hours to a week.

Unfortunately, the doctor does not state whether he has had any cases of failure of this treatment, and if he has, how many. The value of his observations are very much lessened by this omission. In order to give children quinine without their tasting or afterwards vomiting it, he has the doses rolled up in wafers. A wafer is moistened, to make it soft, and then placed in a spoon nearly full of water; the spoon is reached far back upon the tongue, and its contents emptied into the throat; the wafer is swallowed as readily as the water. "The smallest child can swallow the wafer and retain the quinine without vomiting."

*The Prevention of Masturbation.*—This injurious habit is often most difficult to break. Dr. Yellowlees, of Glasgow, speaks of a mode he had tried in a dozen cases, and so far as it had gone he was very much satisfied with the results. The oldest case was eighteen days. The suggestion was founded upon the anatomical fact that the prepuce was anatomically necessary for the erection of the penis. Its anatomical use was to give a cover for the increased size of the organ. If you prevented the prepuce going to that use, you would make erection so painful that it would be practically impossible, and emission therefore extremely unlikely. What he had done was to deal with the prepuce at the very root of the glans, to pierce it with an ordinary silver needle, the ends of which he tied together. He had the case of a lad who was so extremely addicted to masturbation that his mother begged him to do what he could to prevent it. He used the apparatus first in the case of this boy, with most excellent results. He had been masturbating night and day, and he was now so well that he was working as a carpenter. Dr. Yellowlees further said that he had eleven more patients all going about with wires in their penises. There was only one case where he had to take it off, the wire causing a good deal of irritation.—*Med. & Surg. Reporter.*

*The Diet for Gout.*—In a note to the *British Medical Journal*, Dr. John Malcomb writes:

My attention has been given, for many years, to the cause and cure of gout, to which I have a hereditary tendency, my father and grand-father having suffered greatly from this disorder. I soon ascertained that, by attention to diet alone, I could prevent the disease, and for more than thirty years I have steadily adhered to a diet consisting of farinaceous food and fruit, with milk and cream, by which means I have escaped any illness. Among my patients I have found that (when I could not induce them to give up animal food), by partaking only of fish, fowl and rabbit—white meats—their attacks of gout have been of a milder and less frquent character; but in no case have I been able to cure the disease unless I could induce a total abstinance from all flesh food.

*Management of Diphtheria.*—The treatment of this disease is discussed in the *Lancet*, by Dr. Ciattaglia. The following case illustrates his therapeutics:

A. N., a little girl, aged seven, of delicate constitution, complained, on the 2d day of February last, of slight sore throat. On looking at the fauces, I found two diptheritic membranes on both tonsils, of the size of two sixpenny pieces. There was no fever, no constitutional disturbance, not even engorgement of the cervical glands. I confined my prescriptions to simple gargles of phenicated water. Next day the membranes were more diffused, the fauces intensely red and swollen, and the engorgement of the glands increased. Temperature 39° C.; pulse 110; urine normal. I ordered at once fifteen grammes of the chlorate of potash, dissolved in 140 of water, to be given, one tablespoonful every two hours; and I smeared, thrice a day, the membranes with the choral and glycerine. On February 4th, the temperature was 40° C.; pulse 125. The membranes had ceased to spread. The urine presented traces of albumen. I continued the same treatment. In the afternoon I found the fever slightly abated; the fauces perceptibly less swolen and less red; the engorgement of the glands also diminished, and the child was rather relieved. On the 5th the fever had gone; the membranes had almost entirely disappeared from the affected surfaces. I reduced the exhibition of the solution of chlorate of potash to one tablespoonful every three hours, and the application of the choral and glycerine to only twice a day. On the 6th, the improvement had continued. Engorgement of the glands scarcely visible, the urine normal, and the child more calm. On the 8th, the membrane on the right tonsol remained, but was less in extent. On the 10th the cure was complete. All next day I continued the chlorate of potash (one tablespoonful every four hours), and I ceased to apply the chloral and glycerine.

*Hypodermic Injections in Glandular Enlargement.*—The *Practitioner* says that, in a recent paper, Dr. Jacubowitz, of Nagy-Karoly, starts from the principle that no inflammation to which a degenerative action is attributable is occasioned by the injections, but that by this means a solvent of a non-irritating character is brought into direct contact with the glandular tissue. He avoids tincture of iodine, all alcoholic fluids and carbolic acid, and uses instead a weak solution of iodide potassium in the proportion of about one part to thirty of water. He gives two cases in which he obtained extraordinarily successful results. In one case he made a puncture into the most prominent part of a gland which was enlarged to the size of a goose's egg, pushing the needle in obliquely to a considerable distance. After injecting about the fourth of the syringeful a resistance was felt; he then withdrew the needle for a short distance, penetrated a septum on one side, and again injected a quarter part. By repeating this process, he threw in about fifteen grains of the iodide in an ounce of water. The tumor almost immediately became harder, smaller and less painful. After four injections, performed in the course of two days, the tumor gradually dwindled to the size of a hazel-nut, and ultimately vanished altogether. The second case was very similar. Here, however, two dark-blue bodies remained, which were so hard that it seemed to be impossible to inject them. Dr. Jacubowitz, however, injected hypodermically the iodide on to occasions, and with perfect success. Ten injections were required altogether. The small quantity of the iodide required to produce the effects observed is very remarkable.

*A Multiple Antidote.*—Dr. Jeannel, of Paris, has endeavored to answer the question: Is it possible to prepare an agent which shall be officinal, that is susceptible of indefinite preservation, capable of neutralizing, chemically, all poisons in the stomach or in the intestines, or, at least, of transforming them into compounds relatively inoffensive, and then determining their prompt evacuation? He discusses the applicability of the agents suggested by Dorvaul, for this purpose, pointing out what appears to him to be the excellencies and defects of each, and finally proposes the following:

| | *Grammes.* |
|---|---|
| Solution of ferric sulphate, s. g. 1.45 | 100 |
| Water | 800 |
| Calcined magnesia | 80 |
| Washed animal charcoal | 40 |

Let the solution of ferric sulphate be kept in a bottle by itself, and the magnesia and charcoal mixed with the water in another bottle. When required, pour the ferric solution into the other bottle, and shake violently. The mixture should be given repeatedly in doses of fifty to one hundred grammes.

According to Janneal's experiment, this antidote, employed in suitable proportions, renders completely insoluble the preparations of arsenic, zinct, and digitaline, but not the oxide of copper. It leaves in solution notable quantities of mercuric oxide and appreciable quantities of morphia and strychnia, and does not decompose or precipitate either the cyanide of mercury or tartar emetic. It completely saturates free iodine, but acts nly partially on solution of the alkaline hypochlorites.

*Sulphurous Acid in Enteric Fever.*—Thirty cases were treated with sulphurous acid, in doses ranging from three to fifteen drops, in lemonade, every four hours. Only one patient of this number died; this one patient "was a fragile girl, whose life was gradually wasting away with consumption, but she recovered, then relapsed and died." The writer thinks the acid acts as a specific upon the fever poison, arresting at once its further development, and thus exterminates the fever. Amelioration ensues at once, and in a very few days the patients, under the influence of this agent, are convalescent. Within twenty-four hours the tongue becomes moist snd commences to clean; the diarrhœa is speedily arrested, the tympanites subsides, the pulse slows and grows stronger, the digestive faculty speedily asserts itself, and the patient is soon out of danger.—*Chicago Med. Jour.*

*Esmarch's Bandage for Chronic Ulcers.*—Dr. Turney, of Ohio, has employed this bandage in seven cases of ulcers of the leg, one a typical indolent ulcer, with indurated edges, over the internal malleolus of a woman over eighty-five years of age. In six cases the cure was rapid and permanent; in one a portion of the cicatrix gave way, but it was again progressing favorably when the patient disappeared. The bandage was applied firmly from the foot to the knee, once a day, and allowed to remain as long as it could be borne, about ten or fifteen minutes. No other treatment was employed. With each application oxygenated blood takes the place of a fluid unfit for nutrition; the strong pressure effectually overcomes the passive congestion and œdematous infiltration, and the distended vessels, completely relieved of their load of vitiated blood, have an opportunity to recover their lost tonicity.—*Med. Record.*

*Kava-Kava, a Remedy for Gonorrhœa.*—An infusion ot the root of the kav-kava (*Piper methysticum*) has long been a popular remedy for gonorrhœa in the Pacific islands. Three or four scruples, or even more, of the grated root, are macerated for five minutes in 2 pints of water, the whole being frequently shaken up. This water after filtration is given in two doses during the day, before or after meals, and repeated every day until a cure is effected. Twenty minutes after the first dose, a pressing desire to urinate is experienced. The quantity of urine is abundant, and it becomes as limpid and as clear almost as water. The pain that was present during the previous micturilions disappears, and a sensation of comfort is experienced in urinating. A cure requires ten or twelve days. The kava, moreover, acts like a bitter tonic. It is pleasant to take, stimulates the appetite, does not derange the digestive functions, and produces neither diarrhœa nor constipation.—*Gazette Medicale de Paris*, April 1.

*Nasal Catarrh Treated Locally.*—Mr. W. Spencer Watson (*Lancet*, April 22), in referring to Dr. Ferrier's communication, "How to Cure Cold in the Head," speaks very highly of the good effects of tobacco snuff. The first pinch generally excites sneezing, but after the second and third pinch the powder remains in the nostrils, and forms a coating for the mucous membrane. A flow of thin serum then begins, and the sense of fullness and obstruction at once subsides. In a few hours the cold is cured, or at the latest survives only a single night. The snuff should be used freely, and the coating formed by it on the mucous membrane should be left undisturbed for several hours, blowing the nose being meanwhile avoided, until all feeling of "stuffiness" in the nostrils has subsided.

*Priapism.*—Dr. Gibbons, Sr., (*Pac. Med. Jour.*) had derived much more benefit in priapism from veratrum viride than from any other agent. He gives the tincture in large doses, at bedtime—seldom less than twenty drops at a dose. His practice is to increase it by a few drops every night till its effect is observable. He has never failed to control priapism by this treatment.

Dr. Simpson described a case from memory, which occurred in the Bellevue Hospital, (N. Y.) a number of years ago. A man had permanent priapism, which resisted all ordinary treatment. At length there was observed a contraction at the base of the organ, resembling a cord, by which it was constricted, and the blood prevented from returning into the body. An incision was then made on each side, which bled freely, and on the next day the trouble was over.

*Gout, Erythema Nodosum.*—Dr. Routh says erythema nodosum and gout are curable by iodide of potassium.—*Pac. Med. Jour.*

*Removal of Foreign Bodies from the Ear.*—Dr. John Cleland suggests that, in removing foreign bodies from the ear, the point of the probe or needle used for extraction should be placed below the object to be dislodged. By so doing it is placed between two inclined planes, and is readily and easily expelled.—*Phila. Med. Times.*

*Cholera Infantum.*—Dr. Cook, (*Louisville Medical News*) in concluding an article on this disease, says:

"Then to summarize, we will state that acute cases of the disease being cholera morbus, the treatment should be urgent. In the slower forms, in which there is inflammation of the mucous lining of the bowels, avoid astringents, except ergot. Quinine, opium, and ergot should be given, separately or combined, alternated with an occasional laxative, as the case seems to demand. For a child two years of age I should prescribe the following:

R. Quiniæ sulph..................... ..........gr. viij;
Opii tinct....................................gtt. xij:
Ergot fl. ext...................................drs. ss;
Fl. ext. licorice....................... . ....drs. viiss.

Misc. Teaspoonful from three to six hours, according to the emergency of the case.

*Incompatibility of Iodide and Chlorate of Potassium.*—Chlorate of potassium and iodide of potassium are both entirely harmless in suitable doses. Furthermore, these two salts do not react upon each other in solution, even at boiling heat. Yet it has been proved that, when they are administered together, they do combine in the stomach, producing iodate of potassium, which is poisonous. M. Melsens found that dogs colud take the chlorate or iodide, in doses of five to seven grains, with impunity, but that a mixture of the two killed them in a few days, with the symptoms of poisoning by iodate of potassium. This combination must, therefore, be avoided. Indeed, as a general rule, the chlorate is so unstable, and so ready to give up its oxygen, that it cannot safely be combined with any substance capable of oxidation.—*M. S. Bidwell in Am. Journ. Pharm.*

*Sulphate of Cinchonidia.*—E. H. Purdum, M.D., of Hermon, Ill., says that since the early part of 1875 he has used the sulphate of cinchonidia quite extensively, especially in intermittent and remittent fevers, with results quite equal to those following the use of sulphate of quina, and with the further advantage that his patients suffer less from the symptoms of cinchonism. The doses used have corresponded to those of the quina sulphate when used under similar circumstances. The irritation of the stomach does not exceed that following the use of any of the other alkaloids. His experience with its use in the multitude of minor affections, such as slight neuralgia, rheumatic troubles, etc., is very favorable.—*Chicago Med. Jour. & Examiner.*

*Acute Rheumatism.*—Dr. J. W. Futrell (*Louisville Medical News*) says: I begin the treatment by putting one ounce each of Epsom salts, nitrate of potash, and powdered sulphur into one quart of boiling water. This, after being allowed to stand in a covered vessel for six hours, is well strained, and given in doses of one ounce three or four times during the day.

To the swollen and painful joints I apply, by means of cloths, a liniment composed of olive oil, oz.v; chloroform, oz.ij; harts-horn, dr.vj; tincture of aconite root, dr.ij. M. Apply sufficiently often to relieve pain. Should the above means not secure rest at night, I administer a full dose of bromide of potash, and repeat in one hour, if necessary.

I have now used it in quite a number of cases of acute and even violent rheumatism, and have not seen it fail to do good.

*Sea Water in the Treatment of Obesity.*—The *Paris Medicale* discusses the treatment of obesity by the administration of sea-water combined with a residence at the seaside. Sea-water taken internally, acts like diuretic and purgative salts, a remarkable fact being that the diuretic effect increases when the purgative diminishes. The water should be obtained, when possible, from some depth and far from shore. It is then to be left to settle for six or twelve hours, and filtered. It is to be taken three times a day, in doses of a small glassful, or in half that quantity at a time, with fresh water or milk. It is stated as a fact that sea-water thus used facilitates the oxygenation of the blood, and that it hastens the elimination of effete materials. In combination with this treatment, sea-water baths are to be taken, free exercise is to be practiced, and fattening foods avoided.

*Syrupus Cinchonæ Alcoholicus.*—Our English exchanges speak of a new pharmaceutical preparation by Mr. Schacht, well known among British pharmacists: Mr. Schacht's object being to present in a concentrated and at the same time agreeable form, all the virtues of Peruvian bark; to obtain, in fact, a preparation which should be exactly equivalent to the cinchona, minus the wood fibre. At the Pharmaceutical Conference, in 1874, Dr. DeVrij stated that such a preparation was only possible by means of extraction with alcohol. On this basis Mr. Schacht has experimented, and he now offers an alcoholic syrup, which in odor and flavor is a very striking reproduction of the finest cinchono. Each fluid drachm is said to contain the medicinal properties of 20 grains of Peruvian bark.

*Treatment of Pruritus Vulvæ.*—At the meeting of the Harveian Society of London, on the 2d of March, Dr. Wiltshire read a paper on Pruritus Vulvæ in which, following the narration of the causes which may lead or contribute to this distressing affection, the following therapeutic measures were recommended. Being a symptom of different maladies, the course to be pursued in each case will necessarily differ. "Of local applications, borax was the best; and next potent, parasiticides. Ointments disagreed, and were too heating. Glycerine did not always agree. Tar or creosote were often useful. In catamenial itching borax did good. Irritating applications were to be avoided. In diabetic pruritus, solutions of borax and careful drying of the parts were to be adopted. In the pruritus of gout, soap was inferior to oatmeal and water. Bromide of potassium internally and externally did good. Arsenic suited some cases admirably. Bran baths were often useful. A curd formed by adding *liquor plumbi* to milk, was often very agreeable.

*The "Bran Bread" Question.*—It seems we are doomed to eat in an uncertainty as to whether superfine flour is the cause of all our physical woes, as a well-known medical writer in New England maintains, or whether it is, after all, the true form in which to prepare wheat for food. On the latter side, Professor Voit says in a late lecture:

"Men have made the greatest errors in being guided merely by chemical analysis, without experiment on the organism. For instance, it is commonly supposed that bread made of whole flour is much better than that made of well sifted flour without bran, because whole flour contains more nitrogen and more ash. But every experiment on man and animals shows exactly the opposite, viz: that bread made of whole flour makes more fæces and is less utilized, not to mention that with whole flour more ash is taken than is wanted."

*Coal Oil.*—Ed. *New Remedies* has this to say:

Regarding the therapeutic powers of petroleum, although it is not officinal in the pharmacopœias of either the United States, Great Britain or France, it has been used for certain affections since a remote period. The German Pharmacopœia contains it under the name of "Oleum Petræ Italiacum." The Arabs long ago alluded to its emmenagogue and diuretic qualities, to its use in asthma and cough, for the dressing of wounds and the expulsion of worms. Externally they employed it as an embrocation in paralysis, chronic rheumatism, and to relieve chilblains.

Seneca oil is a variety, and Haarlem oil a preparation of petroleum. As a cure for scabies it is thought by many to be superior to sulphur, and in several public institutions in this country it has been used to exterminate lice. Diluted with olive-oil or glycerine, it has been employed as an application in prurigo. Internally it is used as an expectorant in asthma and chronic coughs unattended with inflammation; as a cure for rheumatism; in paralytic affections; as a substitute for copevia in treatment of gonorhœa, and cholera of course. Vesicles may result from its free application to a tender skin, but they are comparatively painless and heal readily.

A New Zealand physiciad has given teaspoonful doses of it every other night with a wine-glassful of water, for the treatment of chronic rheumatism; a little salt, previously dissolved in the mouth, palliating the unpleasant taste. The ordinary dose is ten to thirty drops in some mucilaginous vehicle. In *Griffiths' Universal Formulary*, page 435 (Ed. of 1874), are a variety of formulæ, *e. g.*, "British Oil," embrocations for dropsy, chilblains, frozen limbs, and caronic rheumatism, and anthelmintic (tape-worm) and diuretic mixtures.

*Belladonna in Asthma.*—Dr. Lamadrid says: "To get the good effect of belladonna in asthma, it must be given in heroic doses. I usually apply the tincture of the United States Pharmacopœia,, in doses ranging from twenty to sixty drops. The strength of the tincture differs so much, as commonly kept in the shops, that the size of the dose must be lost sight of, and quantity given regulated by the effect produced. It may be given during the paroxysm with great advantage, best when given before the attack commences. For example, if the patient has nocturnal attacks coming on after midnight, as is usual, give him a dose just before going to bed, and repeat it if necessary to produce sound sleep. He fails to awake at the usual time for the attack to commence, and sleeps on, awakening in the morning very much refreshed and strengthened. The treatment may be repeated night after night, until sufficient time has been had to remove the tendency of the disease to return, either by changing its location, or adopting other requisite treatment, as the case may call for. I could relate several cases to prove the above statements, but will have to omit them for want of space.

"Some times, but not often, belladonna produces dryness of the fauces and delirium. These are indications which show that it should be discontinued and hydrate of chloral should be employed in its stead. It may be used on the principle of belladonna, to produce sleep, and thus ward off the attack."—*Med. Brief.*

*Carbolic Acid in Treatmeut of Whooping Cough.*—Samuel Lee writes to the *Br. Med. Jour.*, Mar. 18, p. 348, that he has used carbolic acid inhalations in both mild and severe forms of whooping cough, and in both with considerable relief. In three cases that have come under his notice in adults, in one of which the malady had continued in spite of all treatment for eight months, attended with hæmoptysis and severe and frequent attacks of coughing, the relief obtained from inhalations of the acid was very marked.

Domingos Carlos has also employed carbolic acid as a last resort in the case of a child, three years of age, which had suffered of whooping cough for two weeks, and upon which all other known remedies had been exhausted. The complaint yielded in two days completely. Other cases since treated in like manner gave equally favorable results. His method of administration is: Acid. Carbol. cryst. 0.25 gm. (4 grs.), Aquæ Aurant. flor. 5.0 gm. (4 scrup), Syrup. Acac. 50.0 gm. (14¼ drs.), S. 4 to 6 teaspoonfuls daily.

*Oil of Sandal-Wood in the Treatment of Gonorrhœa.*—Dr. S. B. Merkel (*Medical Times*) says;

"I am fully persuaded that the oil of sandal-wood possesses a much greater power in restoring to a healthy state the mucous membrane of the urethra than does either cubebs or copaiba. In no case have I ever known it to produce sickness.

"There are objections, I admit, to the use of the oil of sandal-wood, on account of the persistent and disagreeable sensation it leaves in the throat, the irritating action it has on the stomach, and the difficulty of obtaining the pure oil, much of it being adulterated and of inferior quality. The first difficulty is overcome when it is given in the form of a capsule; the second, when it is mixed with a tenth part of the common oil of cinnamon; and the third is to be met by selecting a brand of established purity.

*Treatment of Diabetes Mellitus by Glycerin.*—Dr. Julius Jacobs (*Virchow's Archiv*, v. 65, p. 481) alludes to the futility of all previously lauded means of treatment in this affection, and gives notes of two cases treated by Shultz's method. Shultz maintained that diabetics lose, by excessive excretion of sugar, enormous quantities of respiratory material, and are obliged to give up their fat and protein for this purpose. If glycerin, which is not changed into sugar in the organism, but is transformed directly into carbonic acid and water, be administered, this waste is compensated, or rather vicariously prevented, by the glycerin. Jacobs made use of the following formula:

R. Glycerinæ..............................drs. vi;
Pulv. acid. tartaric.......................sc. iv;
Aq. destillat..............................f oz. xss.—M.

To be taken in twenty-four hours.

Although Jacobs did not obtain very brilliant results from this treatment, yet the patient's improvement was sufficient to encourage further use of the remedy. The usual food was taken.

*Sulpho-Carbolate of Soda in Scarlet Fever.*—Dr. Pirant, *Med. and Surg. Reporter* says: I attended three daughters of L. P., aged seventeen, thirteen and two and a half years, for malignant scarlet fever, with miliary eruption, following diphtheria and severe throat complication. For ten days they were treated with sulphite of soda, sulphite of potassium, carbolic acid, chlorate of potassæ and glycerin, salicylic acid, with acetate of ammonia and glycerin, alternated with quinine and muriated tincture of iron; a nourishing diet, and ice in rubber bags externally. The throats were also swabbed every two or three hours with eight per cent. of carbolic acid in glycerin. They became worse and worse, and when at the point of death, I resorted to the following:

| | |
|---|---|
| R.—Sulpho-carbolate of soda, | dr.iss. |
| Aqua flores aurantii, | oz.j. |
| Syrup. pruni virgin, | oz.iss. |
| Syrup. tolu., | oz.ss. |

Sig.—One teaspoonful every two hours, and half that quantity for the youngest. Also—

| | |
|---|---|
| R.—Acid. carbolic. pur., | dr.iss. |
| Glycerin pur., | oz.j. |

Sig.—Swab the throat with a large camel's hair brush, and continue ice externally.

For ten hours from the above first dose of the mixture great change for the better took place, and the two previously suffocating patients could again breathe easier. With the above remedy, general tonic and supporting treatment the two youngest rapidly convalesced; but the oldest took pneumonia, complicated with severediarrhœa, both of which subsided under tonic, stimulant and sedative treatment and generous diet; and she made a good recovery.

*Fatal Result of Injection of Chloral.*—For the purpose of performing an operation upon both eyes of a male patient, Deneffe and Van Wetter injected 6 grm, (92½ grs.) of chloral into the blood within ten minutes. After anæsthesia had been obtained, the right eye was operated upon, which was accomplished in one minute. Just as they were proceeding to operate upon the other eye, pulse and respiration suddenly ceased; but on immediately applying a current of electricity, they reappeared, and the face assumed a natural redness. But suddenly the apparatus ceases to work, another is not at hand, the pulse and respiration ceases again, and the patient is dead. This case shows that the utmost caution and reliable appliances for restoring animation should never be wanting.—*Pharm. Centralb.* fr. *Arch. d. Pharm.*

*Glycerole of Subacetate of Lead.*—Mr. C. D. Parry furnishes the following as a supplement and improvement to the formula of this preparation, proposed by Mr. Balmano Squire (see our July number, pp. 171 and 174):

I beg to submit a much easier method of making the glycerole of subacetate of lead, invented by Mr. Squire.

Mix equal portions of liq. plumbi subacetatis and glycerine in an open dish, and evaporate by gentle heat until the water is driven off. When the glycerine is reduced to its original bulk, it may be poured into a bottle, filtering not being necessary. Should the strength be objected to, I think it may be increased two or three times, if necessary.—*Pharm. Journ. & Trans.*, May 27.

R. Oil of bitter almonds......................gtt. vj;

Oil of cloves.......................... } aa drs. j;

Oil of cinnamon..................... }

Alcohol ....................................dr. vj.

M. Apply a few drops to the temple and forehead, and afterward rub in equal quantity of water. For Neuralgia.—*Louisville Medical News.*

R. Quiniæ sulph..............................drs. ss;
Ferri sulph. exsicat...........................drs. ss;
Ext. aloes....................................gr. iv;
Ext. belladonnæ...............................gr. iij;
Syr. simp.....................................q. s.

M. Div. in pil. xxx.

S. One pill three times a day in Sciatica, accompanied with Constipation.—*Louisville Medical News.*

*Pimples on the Face.*—A young lady says she has for many years suffered the mortification consequent upon having a bad complexion, with an abundant crop of pimples, which she says was a great annoyance to her. She had tried everything she could hear of without obtaining relief, and she was about to give up in despair, when she fortunately adopted the use of oatmeal in the following manner: Put a little of the meal in a small vessel, pour on cold water, let stand a few minutes, press out through a cloth, and rub the face with it once or twice a day. She says she used it but a few weeks until the effect was almost marvelous. The remedy has one thing to recommend it—it is perfectly harmless, which can be said of very few of the washes which are often sold in the market and so highly recommended. —*Physio-Med. Jour.*

*Hydro-Chloral by the Rectum in the Vomiting of Pregnancy.*—Dr. D. B. Simmons, Chief Surgeon to Ken Hospital, Yokahama, Japan, reports (*Med. Record,* June 1, 1874) four cases of excessive vomiting of pregnancy in which 30-grain doses of chloral morning and evening, administered in mucilage by rectum, afforded marked relief.

We believe that hydro chloral, administered in this manner will relieve most cases of nervous or sympathetic vomiting, where there is no inflammation especially. Even in strangulated hernia, on theoretical grounds, it ought to act well, not only in checking the vomiting, but in producing relaxation.—*Am. Jour. af Med. Science.*

*Muriate of Ammonia in Enlargement of the Spleen.*—Dr. J. Guild, of Tuscaloosa, Ala., (*Med. & Surg. Reporter*) says;

The muriate ammonia, as recommended by Eberle, is the great desideratum. As a resolvent it has in my hands proved entirely efficacious. It will give speedy relief only when given in purgative doses, thirty or forty grains. I make a saturated solution, and give a tablespoonful every two hours. Other very important adjuvants should not be overlooked, such as the iodine ointment, often applied over the left hypochondrium, with an occasional aloetic purge.

*A Sensible Precaution.*—When lunar caustic is used in the oral cavity, and toward the tonsils and larynx, fears may be entertained that the stick may break and cause dangerous symptoms. To make such an unpleasant accident impossible, Dr. Mettenkeimer places the caustic in a little bag of gauze, through the meshes of which the former acts completely, the escape of the stick being effectually prevented. Of course the gauze should be changed at each cauterization, as the meshes are liable to get obstructed by moisture, and even to be destroyed by the caustic.—*Lancet.*

*On the Methods for Detecting Albumen in Urine.*—A. Hilger has subjected the various methods for detecting albumen in urine to a comparative examination, and has come to the conclusion that Bodeker's reaction, viz: precipitation of albumen in acetic acid solution by means of potassium ferrocyanide, deserves the most confidence, especially when other reagents have yielded doubtful results. He advises physicians in such cases to chiefly rely on the following three methods:

1. Test by means of nitric acid.
2. Coagulation by means of acetic acid.
3. Precipitation by means of potassium ferrocyanide in urine, containing acetic acid.

Professor C. Neubauer adds that, according to his experience, the first of these tests is all-sufficient, if conducted in the manner proposed by Heller, namely: by carefully pouring upon a layer of colorless nitric acid a similar layer of previously filtered urine. (See his *Anleit. z. Anal. des Horns*, 7th edition, p. 76.) —*Zeitsch. f. anal. Chem.* 1876, 115.

*Mesmeric Anæsthesia.*—A lady came to my office, after having broken two engagements, to take an anæsthetic, and said, "Have you any objections to extracting teeth for me while under the influence of mesmerism? Professor W. offers to mesmerize me." I gave my consent. He took a position in front of the patient, and after a few gentle strokes of his hands over her face, motioned to me that all was ready. Six teeth were extracted before she left the chair. She expressed herself as not having felt the least particle of pain.

I do not remember of ever having had so timid and nervous a patient. While making the preliminary examination she would not allow me to use an excavator.—J. C. Henry in *Dental Cosmos.*

"*Universal Disinfecting Powder.*"—The *British Med. Jour.* notices a disinfecting powder, made by Messrs. Ledger & Co., which Mr. Wanklyn, a public analyst, says has the following composition: "The powder contains 70 per cent. of mixed chloride of sodium and chloride of calcium, and about 6 per cent. of anhydrous sulphate of zinc (equal to about 12 per cent. of hydrated sulphate), a little insoluble matter, and 15 per cent. of moisture."

The powder is comparatively non-poisonous; will stop putrefaction at once, will absorb the volatile ammoniated products of decomposition, and render them innocuous; it will act in the same way with sulphuretted hydrogen, and thus it fulfils all the functions of an efficient disinfectant. It is highly soluble: 90 per cent. of it being completely dissolved in water; and it is applicable in either powder or solution.

*Milk as a Vehicle for Bromide of Potassium.*—Dr. A. K. Minich writes to the Philadelphia *Medical Times* that a patient suffering from alcoholism stoutly refused to take bromide of potassium or any other "confounded medicine." Twenty grains were dissolved in a glass of milk, which he drank readily. "Since then," says Dr. Minich, "I find that twenty grains are entirely disguised by one ounce of milk. I have also found milk a very useful liquid to 'wash down' salicylic-acid wafers. It has always in my hands prevented the burning in the stomach which is so often produced when the acid is given in large and oft-repeated doses."

*Van Swieten's Liquor.*—

| | |
|---|---|
| Corrosive sublimate, | 1 part by weight. |
| Alcohol, | 100 parts by weight. |
| Water, | 900 parts by weight. |

Dissolve. A tablespoonful night and morning in a glass of water, in milk or in decoction of sapsaparilla.—*Druggist Circular and Chemical Gazette.*

*Alum, Tannin, and Oxide of Zinc in Sticks.*—When these substances are to be carried into the neck or body of the uterus they are liable to break and become troublesome. It has been attempted to incorporate them with glycerine, but unsuccessfully. Mr. Duquesnel is using gutta percha, which he mixes with the medicinal substances by means of heat. While the combination is still hot, it is rolled into cylinders a few lines thick, which harden on cooling. It remains to be proved that the astringent effects are not hindered by the gutta percha.—*New Remedies.*

*Powder for Producing Ozone.*—In order to produce artificial ozone, Mr. Lender makes use of equal parts of peroxide of manganese, permanganate of potassium, and oxalic acid. When this mixture in placed in contact with water, ozone is quickly generated. For a room of medium size, two spoonfuls of this powder placed on a dish and occasionally diluted with water would be sufficient. The ozone develops itself; it disinfects the surrounding air without producing cough.—*Medical Press and Circular.*

*Extract of Beef.*—Bouchardat warns against incautious use of this extract, under the mistaken notion that an increase of dose will be followed by a corresponding increase of benefit. Both he and Stuart Cooper have shown that large doses of the extract are quite injurious. He further asserts that it cannot at all be compared to meat-juice (expressed in the cold from raw meat as a strength giver.—*Bull. Therap.*

*Camphorated Ether in Erysipelas.*—According to Cavazzani, a preparation consisting of camphor and tannin, of each one part, and ether eight parts, is an excellent application, even in cases of phlegmonous erysipelas. It is also very efficacious in burns of the first and second degrees; the heat disappears rapidly, and blisters do not form. It should be applied every three hours. —*Medical Record.*

*Loomis' Tonic.*—

R. Sol. Quin. Sulph. [grs. 15 in 1 fl. oz] fl. oz. 2
Tr. Ferri. Chlor........................................fl. dr. 4
Spts. Chloroformi....................................fl. dr. 6
Glycerinæ....................................q. s. ad oz. 4

M. Teaspoonful.

